# Tinnitus prevalence and characteristics in the United States: insights from a cross-sectional analysis of the 2019–2022 Apple Hearing Study cohort

**DOI:** 10.1186/s12889-026-27048-2

**Published:** 2026-03-19

**Authors:** Ying Tang, Xin Zhang, Lauren M. Smith, Abas Shkembi, Glenn E. Green, Richard L. Neitzel

**Affiliations:** 1 Department of Environmental Health Sciences, School of Public Health, University of Michigan, 1415 Washington Heights, Ann Arbor, MI 48109-2029 U.S.; 2 Department of Otolaryngology–Head and Neck Surgery, University of Michigan, Michigan Medicine, Ann Arbor, MI U.S.

**Keywords:** Tinnitus, Prevalence, Hearing health, Cross-sectional study, Apple Hearing Study

## Abstract

**Background:**

Tinnitus is a complex condition with significant heterogeneity in its presentation, and its risk factors remain poorly characterized, posing challenges for prevention, diagnosis, and treatment. This study aimed to assess the prevalence, characteristics, and risk factors of tinnitus in the United States (U.S.) using large-scale survey data.

**Methods:**

We conducted a cross-sectional analysis of 125,252 volunteer adults (≥ 18 years) enrolled in the Apple Hearing Study, a nationwide app-based cohort of iPhone users in the U.S. (November 2019–November 2022). The outcomes were the weighted prevalence of any tinnitus and bothersome tinnitus, measured using self-reported tinnitus frequency, duration, awareness, loudness, and interference with hearing. Age-adjusted and multivariable logistic regression models were applied to analyze the odds ratios of self-reported potential risk factors on tinnitus, and a weighted decision tree identified the strongest predictors of bothersome tinnitus.

**Results:**

The estimated weighted national prevalence of any tinnitus was 30.8% (95% Confidence Interval [CI]: [30.3%, 31.2%]) and bothersome tinnitus was 11.6% (95% CI: [11.3%, 11.9%]). Controlling for age, sex, race/ethnicity, and other sociodemographic characteristics, self-rated hearing ability was the strongest risk factor for any tinnitus (odds ratios of 4.52 [95% CI: 4.03–5.06] and bothersome tinnitus 8.88 [95% CI: 7.52–10.49], comparing poor to excellent hearing). The odds of both types of tinnitus increased with age, peaking in the 60–64 age group (2.01 [95% CI: 1.77–2.28] for any tinnitus and 2.72 [95% CI: 2.24–3.92] for bothersome tinnitus) after adjusting for the same set of variables. Non-Hispanic Whites had higher odds of any and bothersome tinnitus compared to other race/ethnicities. A reported history of occupational noise exposure was associated with higher odds of any and bothersome tinnitus.

**Conclusions:**

Approximately 3 in 10 U.S. adults are estimated to experience any tinnitus, and about 1 in 10 affected by bothersome tinnitus. Tinnitus is associated with worse self-rated hearing ability, age, race/ethnicity, and a history of workplace noise. These results align with prior epidemiological estimates and demonstrate the feasibility of using app-based platforms to collect large-scale, high-quality hearing health data.

**Supplementary Information:**

The online version contains supplementary material available at 10.1186/s12889-026-27048-2.

## Background

Tinnitus is the perception of sound without the presence of external sound [1, 2], typically experienced as ringing, whistling, or buzzing which can vary in intensity, frequency and duration [3]. Hearing is not a necessary condition for tinnitus, which even deaf individuals can experience [4, 5], however, hearing loss and noise exposure are well recognized risk factors for tinnitus in most cases [6, 7]. Frequent and longstanding tinnitus can significantly impact quality of life, leading to sleep disturbances, social isolation, mood alterations, and an overall decline in health and well-being [8–10].

Tinnitus prevalence varies substantially across studies due to differing definitions based on duration [11], frequency [7], and perceived distress [12]. The presence of any tinnitus is commonly defined by episodes longer than five minutes, and the estimated U.S. prevalence is around 25% [7]. In contrast, bothersome tinnitus (defined as interfering with a person’s life sufficiently to cause worry, annoyance or upset) [11] affects about 6% of European Union residents. Estimates of severe tinnitus—typically operationalized using validated instruments (e.g., Tinnitus Handicap Inventory [THI]) [13] that assess tinnitus-related functional impairment, such as effects on sleep, concentration, and overall quality of life [14], is 3.3% in North America [15].

The risk factors for tinnitus are incompletely understood [1, 16]. Tinnitus increases with age [7, 17], and is strongly associated with hearing loss independent of age [11, 18], especially among individuals with severe hearing loss (> 65 decibel hearing level [dB HL]) [12]. There are mixed results regarding sex, with some studies showing higher tinnitus prevalence among males [13, 19] or females [12], and a pooled analysis of > 300,000 adults showing no significant difference between sexes [15]. Significant associations have been found between race/ethnicity and tinnitus, with non-Hispanic Whites experiencing a higher prevalence than non-Hispanic Blacks and Hispanics [7]. Finally, noise exposure – and especially occupational noise – play a role in the development of tinnitus [20, 21].

In this analysis, to our knowledge the largest on tinnitus in the U.S. to date, we offer a robust understanding of tinnitus and potential risk factors.

## Methods

### Study population

Initiated in 2019, the Apple Hearing Study (AHS) is a nationwide prospective study with a primary objective to investigate how health, behavior, and sound exposure impact hearing over time [22]. For this analysis, we started with 186,931 participants 18 years of age and older who provided informed consent and enrolled in the study using Research app on their iPhone between November 2019 and November 2022. Participants were asked five tinnitus questions (see Additional file 1) in a baseline survey: two on general tinnitus experience (i.e., frequency and duration) and three on tinnitus experience in the past two weeks (i.e., awareness, loudness and interferences with hearing). Participants who did not answer the tinnitus frequency question (*N* = 903) or the general tinnitus duration question (*N* = 29,942) were excluded (see Additional file 2). Participants missing any of four self-reported demographic values (age, sex, race/ethnicity, and education) were also excluded (*N* = 30,834). These exclusions resulted in 125,252 participants being included in the analyses.

### Survey questions and operational definitions of tinnitus

Our primary outcomes were the presence of “any tinnitus” and “bothersome tinnitus” based on responses to the five tinnitus questions. Because the Apple Hearing Study was not designed as a tinnitus-specific study, standardized tinnitus instruments were not included. Tinnitus and bothersome tinnitus were therefore defined using the available tinnitus-related questions in the baseline survey, with criteria selected to align as closely as possible with those commonly used in the literature.

In our analysis “any tinnitus” refers to individuals who reported having experienced tinnitus for at least a few times a year and it lasts at least a few minutes at each occurrence. Participants were classified as having “any tinnitus” if they reported that a typical tinnitus episode lasted “a few minutes to an hour”, “hours to days” or “it is constant” to the question, “How long does your typical episode of tinnitus (sound like ringing, whistling, buzzing, or roaring in your ears) last?” This definition was intended to approximate commonly used tinnitus criteria, namely “tinnitus lasting more than five minutes at a time in the past 12 months” [13]. All other response categories (i.e., “less than a few second” and “a few seconds to a minute*”)* were included in the non-tinnitus group.

**Fig. 1:**
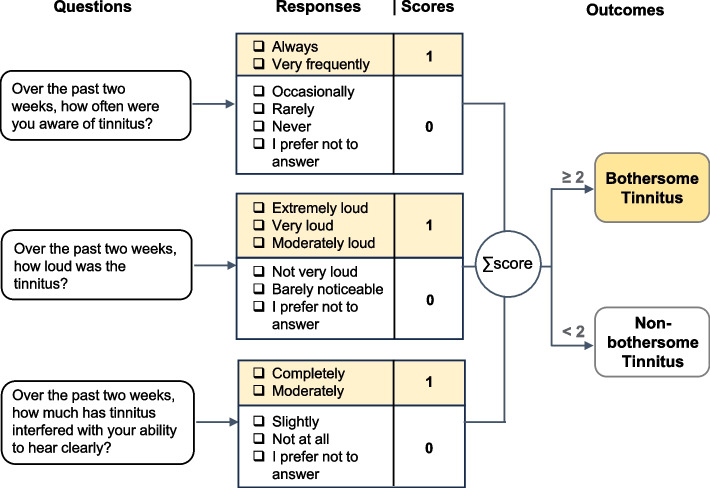
Flowchart for identifying bothersome tinnitus. For each of the three questions, tinnitus participants score 1 if they selected a response from the highlighted options, otherwise 0. Participants with a total score of 2 or more out of 3 were classified as having bothersome tinnitus; those with fewer than 2 points were classified as not having bothersome tinnitus

“Bothersome tinnitus” refers to individuals with “any tinnitus” who have recently experienced tinnitus that was frequent, loud, and/or interfered with hearing, with at least two of these three characteristics being present. Specifically, among participants with any tinnitus, those classified as having bothersome tinnitus were identified based on responses to the three questions about tinnitus experience over the past two weeks: “How often were you aware of tinnitus?”, “How loud was the tinnitus?”, and “How much has tinnitus interfered with your ability to hear clearly?”. Responses for each question were dichotomized to 0 or 1, and “bothersome tinnitus” was assigned if the sum of the three responses was 2 or more (Fig. 1). This approach infers the degree to which tinnitus is bothersome from its frequency, loudness, and interference with hearing, rather than relying on a direct question about subjective bother.

### Statistical analysis

#### Descriptive statistics

Analyses were conducted using RStudio v3.6 and v4.1.2 (R Core Team). The non-random, self-selected AHS population disproportionately consists primarily of White males and is younger and more highly educated compared to the U.S. general population. Therefore, to estimate non-biased prevalences, we assigned each participant a weight based on age (18–24, 25–34, 35–44, 45–54, 55–64 and 65 + years), sex (female and male), race/ethnicity (non-Hispanic White, non-Hispanic Black, Hispanic, non-Hispanic Asian and other) and education (below bachelor’s, bachelor’s, above bachelor’s) distribution in comparison to nationwide estimates in the American Community Survey (ACS) 2018–2022 [23], using the ‘anesrake’ package for raking sample weighting [24]. Weighted prevalences for “any tinnitus” and “bothersome tinnitus” were estimated using the `survey’ package. Prevalences were summarized by various sociodemographic characteristics, self-rated hearing ability, and years of occupational noise exposure, with 95% confidence intervals reported (using the `survey’ package).

#### Regression analyses

To assess sociodemographic and risk factor differences in both tinnitus outcomes we ran age-adjusted weighted logistic regression models for each factor, as well as a single weighted multivariable logistic regression that includes all sociodemographic characteristics and risk factors together. Sociodemographic characteristics of interest were sex, race/ethnicity, education, employment status, marital status, perceived socioeconomic status [25], U.S. region, household count and population of their hometown (see Additional file 1). Self-reported risk factors included their self-rated hearing ability, and their years of occupational noise exposure (see Additional file 1). We relied on weighted age-adjusted odds ratios (AORs) and multivariable-adjusted odds ratios (MORs) to interpret the magnitude of associations, as these provide more meaningful insights than p-values when large sample sizes (e.g., n > 10,000) make even small differences statistically significant [26]. This regression analysis was conducted using the ‘survey’ package. All regression analyses used tenfold cross-validation to enhance result robustness.

#### Decision tree

Weighted decision tree analysis was performed to determine the strongest sociodemographic characteristics and risk factors for bothersome tinnitus (yes/no), where “no” included both non-bothersome tinnitus and no tinnitus, and to assess internal model performance. Decision trees overcome the limitation of traditional regression models (which assume that all model covariates are independent) by accounting for dependence and interactions among all covariates, making them appropriate for drawing inferences on the multiple risk factors of tinnitus [27]. The weighted decision tree was constructed using the `rpart` package to predict bothersome tinnitus (yes or no) based on all the previously mentioned sociodemographic characteristics and risk factors. To identify the single best tree, the Gini index was employed to identify the optimal splits, maximizing Gini gain at each step. To prevent overfitting, tenfold cross-validation was applied, and the tree was pruned by selecting the minimum average cross-validated error rate, which helped reduce complexity and improve interpretability. A cost function was used to address class imbalance, assigning a cost of 12 for misclassifying the minority class (bothersome tinnitus) to enhance model sensitivity. Model performance was evaluated using the area under the receiver operating characteristic curve (AUC), prioritizing the algorithm with the highest sensitivity alongside a high AUC.

## Results

Among the 125,252 participants, 32,943 reported experiencing any tinnitus, yielding an estimated weighted prevalence of 30.8% (95% CI: [30.3%–31.2%]) (26.3% actual), and 9,903 participants experienced bothersome tinnitus, with an estimated weighted prevalence of 11.6% (95% CI: [11.3%, 11.9%]) (7.9% actual). Participant characteristics and weighted prevalence estimates of any and bothersome tinnitus are shown in Table 1.

**Table 1:** Characteristics and weighted prevalence estimates of any and bothersome tinnitus

Characteristics	No. AHS subjects	Any Tinnitus		Bothersome Tinnitus	
		Unweighted, %	Weighted, % (95% CI)	Unweighted, %	Weighted, % (95% CI)
Overall	125252^a^	26.3	30.8 (30.3, 31.2)	7.9	11.6 (11.3, 11.9)
Age, years					
18–24	20,904	19.3	18.5 (17.8, 19.2)	4.4	4.6 (4.3, 5.0)
25–29	17,136	19.5	19.0 (18.1, 19.9)	4.5	5.5 (5.0, 6.1)
30–34	18,395	20.8	20.6 (19.7, 21.6)	4.9	6.0 (5.5, 6.6)
35–39	17,619	23.5	21.8 (20.8, 22.7)	6.4	7.0 (6.4, 7.6)
40–44	14,708	26.4	24.7 (23.6, 25.8)	7.8	8.5 (7.8, 9.2)
45–49	10,780	30.7	28.5 (27.2, 29.7)	10.0	10.2 (9.4, 11.0)
50–54	8939	35.7	32.9 (31.5, 34.3)	12.5	13.4(12.3, 14.4)
55–59	5968	39.8	38.3 (36.8, 39.9)	15.0	16.6 (15.4, 17.9)
60–64	4104	44.2	43.4 (41.4, 45.4)	18.0	19.6 (18.2, 21.2)
65–69	3316	45.7	45.6 (43.9, 47.3)	19.1	19.6 (18.2, 21.0)
70 +	3383	45.3	45.7 (43.9, 47.5)	17.1	17.7 (16.3, 19.1)
Sex					
Female	49,954	25.4	28.1 (27.5, 28.7)	7.8	10.7 (10.2, 11.1)
Male	75,298	26.9	33.5 (32.9, 34.1)	8.0	12.6 (12.1, 13.0)
Race/ethnicity					
Non-Hispanic White	90,646	28.9	37.6 (37.0, 38.1)	8.7	14.6 (14.1, 15.0)
Non-Hispanic Black	5428	16.6	17.5 (16.3, 18.7)	4.9	5.7 (5.0, 6.5)
Hispanic	14,138	19.3	21.5 (20.6, 22.4)	5.8	7.7 (7.1, 8.3)
Non-Hispanic Asian	6716	14.2	17.1 (15.7, 18.5)	2.8	4.2 (3.4, 5.1)
Other^b^	8324	26.1	33.0 (31.3, 34.7)	8.5	14.1 (12.7, 15.5)
Education					
< High school	2630	22.9	22.9 (20.7, 25.1)	9.7	10.3 (8.7, 11.9)
High school or GED	15,586	23.0	24.6 (23.7, 25.6)	8.2	10.0 (9.3, 10.7)
> High school	107,036	26.9	32.4 (31.9, 32.9)	7.8	12.0 (11.6, 12.3)
Employment					
Employed	94,119	25.3	27.6 (27.1, 28.1)	7.0	9.5 (9.2, 9.8)
Unemployed	6565	24.6	24 (22.5, 25.5)	8.5	9.3 (8.3, 10.4)
Retired	6492	45.6	46.7 (45.3, 48.0)	19.2	19.9 (18.8, 21.0)
Student	10,368	19.2	18.5 (17.5, 19.6)	4.1	4.4 (3.9, 5.0)
Other	6968	34.3	35.4 (33.8, 36.9)	14.7	17.1 (15.8, 18.4)
Marital status					
Never married	39,621	20.7	20.9 (20.3, 21.6)	4.9	6.1 (5.7, 6.5)
Married or couple	70,963	28.4	34 (33.4, 34.6)	8.7	13.1 (12.7, 13.6)
Divorced, widowed or separated	13,684	32.4	35 (33.8, 36.1)	12.5	14.7 (13.8, 15.6)
Socioeconomic status^c^					
Higher (0 ~ 5)	99,275	26.0	31.4 (30.9, 31.9)	7.5	11.6 (11.2, 12.0)
Lower (6 ~ 9)	25,796	27.3	28.7 (27.9, 29.5)	9.6	11.6 (11.0, 12.2)
Region					
Northeast	20,736	23.6	27.7 (26.6, 28.7)	6.3	9.8 (9.1, 10.6)
Midwest	25,671	27.4	32.6 (31.6, 33.6)	8.1	12.0 (11.3, 12.7)
South	43,781	26.5	30 (29.3, 30.7)	8.6	11.9 (11.4, 12.4)
West	34,762	26.9	32.3 (31.5, 33.1)	7.8	11.9 (11.3, 12.5)
Puerto Rico and other^d^	300	20.0	25.8 (18.3, 33.3)	7.3	8.7 (4.0, 13.3)
Household count					
One	17,042	26.9	33.1 (31.9, 34.3)	7.8	12.5 (11.6, 13.4)
Two to four	90,087	26.5	31.5 (31.0, 32.0)	7.9	12.0 (11.6, 12.3)
Five or more	17,423	24.7	24.3 (23.3, 25.3)	7.9	8.7 (8.1, 9.4)
Population of town					
< 10 k	16,510	29.3	34.6 (33.4, 35.7)	10.1	14.1 (13.2, 15.0)
10 k ~ 1,000 k	80,133	26.6	31.4 (30.9, 31.9)	7.9	11.8 (11.4, 12.2)
> 1,000 k	26,215	24.1	27.2 (26.3, 28.1)	6.7	9.6 (8.9, 10.2)
Self-rated hearing ability^e^					
Excellent	22,301	14.9	15.8 (15.0, 16.6)	2.8	3.9 (3.5, 4.3)
Very good	41,488	20.0	21.9 (21.2, 22.6)	3.6	5.2 (4.8, 5.6)
Good	37,050	29.1	32.7 (31.9, 33.5)	7.8	10.7 (10.1, 11.2)
Fair	19,051	40.7	45.5 (44.5, 46.6)	16.9	21.4 (20.4, 22.3)
Poor	5255	52.2	56.1 (54.2, 58.0)	31.5	35.1 (33.3, 37.0)
Occupational noise exposure duration^f^					
0 year	65,482	23.6	28.3 (27.7, 28.9)	5.6	9.1 (8.7, 9.5)
> 0 to 4 years	29,306	27.0	28.8 (28, 29.7)	8.4	10.5 (9.9, 11.0)
5 to 10 years	10,700	32.4	35.1 (33.7, 36.5)	12.5	15.9 (14.8, 17.1)
11 to 15 years	3591	36.8	40.1 (37.7, 42.6)	15.3	18.7 (16.7, 20.7)
> 15 years	5135	44.0	48.2 (46.3, 50.0)	21.6	24.7 (23.1, 26.3)

Except for age, sex, education, and race/ethnicity, other sociodemographic and risk variables may not sum to 125,252 due to skipped responses (92–2387) or missing values (7–181), excluding ‘occupational noise exposure duration,’ which had 10,783 missing values due to change of survey question logic between survey V1 and V2 (see Additional file 2)

^a^The total number of AHS participants categorized by age, sex, race/ethnicity, and education is 125,252. Counts for other variables do not add up to this total because, except for occupational noise exposure duration, 700 to 2394 participants in each sociodemographic group selected 'I prefer not to answer.' Additionally, due to the survey's logic design, occupational noise exposure duration has 11,038 missing values

^b^"Other" includes participants who identify as a different race/ethnicity or as multiracial

^c^In the AHS demographic survey, participants rated their socioeconomic status using the MacArthur Scale of Subjective Social Status, a ladder scale from 0 to 9 in the U.S., where 0 represents the "best off"—those with the most money, most education, and most respected jobs—and 9 represents the "worst off”

^d^“other” includes all the other U.S. territories

^e^Participants were asked, “How would you rate your hearing ability?” with response options: “Excellent”, “Very good”, “Good”, “Fair”, “Poor”, and “I prefer not to answer”

^f^Participants who answered “Yes” to working in a loud workplace were then asked, “How many years have you worked in a loud workplace?” with response options: Less than 5 years, 5–10 years, 11–15 years, More than 15 years, and I prefer not to answer

### Tinnitus prevalence

The weighted prevalence of any and bothersome tinnitus was higher in older adults, peaking in the 70 + age group at 45.7% (95% CI: [43.9%–47.5%]) and in the 60–64 age group at 19.6% (95% CI: [18.2%–21.0%]), respectively (Table 1). Males had higher prevalence than females for both conditions. Non-Hispanic Whites had the highest estimated prevalence of any tinnitus among all race/ethnics group at 37.6% (95% CI: [37.0%–38.1%]) and bothersome tinnitus at 14.6% (95% CI: [14.1%–15.0%]).

Participants who rated their hearing as poor had a much higher prevalence of any tinnitus (56.1%, 95% CI: [54.2%–58.0%]) and bothersome tinnitus (35.1%, 95% CI: [33.3%–37.0%]) compared to those who reported excellent hearing (15.8% (95% CI: [15.0%, 16.6%]) and 3.9% (95% CI: [3.5%–4.3%]), respectively). Participants with a longer history of occupational noise exposure showed higher prevalence; after more than 15 years of occupational noise exposure, 48.2% (95% CI: [46.3%–50.0%]) reported any tinnitus and 24.7% (95% CI: [23.1%–26.3%]) reported bothersome tinnitus.

### Characterizing tinnitus

Among the 32,943 participants with any tinnitus, 46.4% reported experiencing daily and 37.6% reported that their tinnitus episode was constant. Over the prior two weeks, 17.2% were always aware of their tinnitus, 2.5% described it as extremely loud, and 2.2% reported that it completely interfered with their ability to hear (Table 2).

**Table 2:** Unweighted tinnitus evaluation questions and responses, *N* = 32,943

Tinnitus general evaluation among participants with any tinnitus			
Question	Response	No. AHS subjects	Percentage, %
How often do you hear tinnitus?	Up to a few times a day	15,276	46.4
	Up to a few times a week	5725	17.4
	Up to a few times a month	4724	14.3
	Up to a few times a year	7218	21.9
How long does your typical episode of tinnitus last?	It is constant	12,400	37.6
	Hours to days	4728	14.4
	A few minutes to an hour	15,815	48.0
Tinnitus evaluation over the past two weeks among participants with any tinnitus			
Question	Response	No. AHS subjects	Percentage, %
How often were you aware of tinnitus?	Always	5675	17.2
	Very frequently	6446	19.6
	Occasionally	9591	29.1
	Rarely	5801	17.6
	Never	5141	15.6
How loud was the tinnitus?	Extremely loud	836	2.5
	Very loud	2742	8.3
	Moderately loud	10,903	33.1
	Not very loud	10,441	31.7
	Barely noticeable	2580	7.8
	Never	5141	15.6
How much has tinnitus interfered with your ability to hear clearly?	Completely	719	2.2
	Moderately	3980	12.1
	Slightly	8953	27.2
	Not at all	13,826	42.0
	Never	5141	15.6

Of the 32,943 participants with any tinnitus, 265 had missing responses for tinnitus awareness, loudness, and interference in the past two weeks due to changes in their reported tinnitus frequency from 'never' to another category. The estimated percentages account for these missing values, although they are not explicitly shown in the table

### Risk factors for tinnitus

Table 3 summarizes the age-adjusted odds ratio (AOR) and multivariable odds ratio (MOR) for any and bothersome tinnitus, with the multivariable models adjusting for key covariates including age, sex, race/ethnicity, occupational noise exposure, and socioeconomic factors. All older age groups had higher AORs than the 18–24 reference group, peaking at 3.72 (95% CI: 3.40–4.07) for any tinnitus in the 70 + age group and 5.01 (95% CI: 4.39–5.72) for bothersome tinnitus in the 65–69 age group. In the multivariable logistic regression model, which also accounted for self-rated hearing ability, the highest MOR was among the 60–64 age group for both any tinnitus and bothersome tinnitus.

**Table 3:** Age-adjusted and multivariable odds ratios of any and bothersome tinnitus

Characteristics	Any Tinnitus				Bothersome Tinnitus			
	Age-adjusted OR^a^ (95% CI)		Multivariable OR^b^ (95% CI)		Age-adjusted OR^a^ (95% CI)		Multivariate OR^b^ (95% CI)	
Age, years								
18–24	Ref		Ref		Ref		Ref	
25–29	1.04 (0.96, 1.12)		0.94 (0.85, 1.03)		1.20 (1.04, 1.38)	*	1.03 (0.87, 1.21)	
30–34	1.15 (1.06, 1.24)	***	1.00 (0.91, 1.10)		1.32 (1.15, 1.52)	***	1.07 (0.90, 1.26)	
35–39	1.23 (1.14, 1.33)	***	1.03 (0.94, 1.14)		1.55 (1.36, 1.78)	***	1.22 (1.03, 1.44)	*
40–44	1.45 (1.34, 1.57)	***	1.14 (1.03, 1.26)	*	1.91 (1.68, 2.18)	***	1.39 (1.17, 1.65)	***
45–49	1.76 (1.62, 1.91)	***	1.30 (1.17, 1.44)	***	2.33 (2.04, 2.66)	***	1.58 (1.33, 1.88)	***
50–54	2.17 (1.99, 2.36)	***	1.55 (1.39, 1.72)	***	3.18 (2.78, 3.62)	***	2.15 (1.81, 2.55)	***
55–59	2.75 (2.52, 2.99)	***	1.77 (1.58, 1.98)	***	4.10 (3.59, 4.68)	***	2.45 (2.05, 2.93)	***
60–64	3.39 (3.07, 3.74)	***	2.01 (1.77, 2.28)	***	5.0 (4.34, 5.76)	***	2.72 (2.24, 3.29)	***
65–69	3.70 (3.38, 4.04)	***	1.89 (1.66, 2.14)	***	5.01 (4.39, 5.72)	***	2.54 (2.09, 3.09)	***
70 +	3.72 (3.40, 4.07)	***	1.67 (1.45, 1.93)	***	4.42 (3.86, 5.07)	***	1.80 (1.45, 2.24)	***
Sex								
Female	Ref		Ref		Ref		Ref	
Male	1.11 (1.07, 1.16)	***	1.15 (1.09, 1.21)	***	1.02 (0.96, 1.09)		0.98 (0.91, 1.06)	
Race/ethnicity								
Non-Hispanic White	Ref		Ref		Ref		Ref	
Non-Hispanic Black	0.46 (0.42, 0.51)	***	0.54 (0.49, 0.60)	***	0.50 (0.43, 0.58)	***	0.61 (0.52, 0.72)	***
Hispanic	0.63 (0.59, 0.67)	***	0.66 (0.61, 0.70)	***	0.73 (0.66, 0.81)	***	0.77 (0.69, 0.86)	***
Non-Hispanic Asian	0.44 (0.39, 0.49)	***	0.46 (0.41, 0.52)	***	0.35 (0.28, 0.43)	***	0.42 (0.33, 0.53)	***
Other^c^	0.97 (0.89, 1.06)		0.98 (0.89, 1.07)		1.18 (1.03, 1.34)	*	1.19 (1.03, 1.37)	*
Education								
< High school	Ref		Ref		Ref		Ref	
High school or GED	1.01 (0.88, 1.17)		1.05 (0.89, 1.23)		0.85 (0.7, 1.05)		1.00 (0.79, 1.28)	
> High school	1.13 (0.99, 1.30)	•	1.26 (1.07, 1.47)	**	0.75 (0.61, 0.9)		1.01 (0.80, 1.27)	
Employment								
Employed	Ref		Ref		Ref		Ref	
Unemployed	0.96 (0.87, 1.05)		0.96 (0.87, 1.06)		1.18 (1.03, 1.36)	*	1.12 (0.96, 1.31)	
Retired	1.28 (1.17, 1.40)	***	1.25 (1.14, 1.37)	***	1.43 (1.27, 1.61)	***	1.33 (1.17, 1.51)	***
Student	0.95 (0.87, 1.04)		1.07 (0.97, 1.18)		0.86 (0.72, 1.02)		1.02 (0.85, 1.23)	
Other	1.40 (1.29, 1.51)	***	1.32 (1.21, 1.44)	***	1.90 (1.71, 2.10)	***	1.54 (1.37, 1.74)	***
Marital Status								
Never married	Ref		Ref		Ref		Ref	
Married or couple	1.22 (1.15, 1.29)	***	1.09 (1.02, 1.16)	*	1.32 (1.19, 1.45)	***	1.17 (1.05, 1.32)	*
Divorced, widowed or separated	1.18 (1.09, 1.27)	***	1.04 (0.95, 1.13)		1.39 (1.24, 1.57)	***	1.14 (1.0, 1.30)	•
Socioeconomic Status^d^								
Higher (0 ~ 5)	Ref		Ref		Ref		Ref	
Lower (6 ~ 9)	1.24 (1.18, 1.30)	***	1.12 (1.05, 1.18)	***	1.51 (1.39, 1.63)	***	1.18 (1.08, 1.29)	***
Region								
Northeast	Ref		Ref		Ref		Ref	
Midwest	1.31 (1.22, 1.41)	***	1.16 (1.07, 1.26)	***	1.3 (1.16, 1.45)	***	1.12 (0.99, 1.26)	
South	1.16 (1.09, 1.24)	***	1.13 (1.05, 1.22)	**	1.29 (1.17, 1.43)	***	1.21 (1.09, 1.36)	**
West	1.24 (1.16, 1.33)	***	1.22 (1.13, 1.32)	***	1.23 (1.11, 1.37)	***	1.18 (1.05, 1.33)	**
Puerto Rico and other^e^	0.96 (0.63, 1.46)		0.96 (0.58, 1.60)		0.91 (0.48, 1.74)		0.83 (0.39, 1.80)	
Household Count								
One	Ref		Ref		Ref		Ref	
Two to four	1.07 (1.0, 1.14)	•	1.0 (0.92, 1.08)		1.1 (1.0, 1.21)	•	1.05 (0.93, 1.18)	
Five or more	1.03 (0.95, 1.12)		0.97 (0.88, 1.07)		1.15 (1.01, 1.30)	*	1.03 (0.89, 1.20)	
Population of Town								
< 10 k	Ref		Ref		Ref		Ref	
10 k ~ 1,000 k	0.89 (0.83, 0.94)		0.97 (0.9, 1.04)		0.84 (0.77, 0.92)		0.96 (0.87, 1.06)	
> 1,000 k	0.75 (0.69, 0.81)		0.92 (0.85, 1.0)	•	0.69 (0.62, 0.77)		0.89 (0.79, 1.01)	•
Self-rated hearing ability^f^								
Excellent	Ref		Ref		Ref		Ref	
Very good	1.42 (1.31, 1.53)	***	1.36 (1.25, 1.47)	***	1.26 (1.08, 1.46)	**	1.27 (1.08, 1.49)	**
Good	2.30 (2.14, 2.48)	***	2.12 (1.96, 2.30)	***	2.56 (2.22, 2.94)	***	2.44 (2.10, 2.83)	***
Fair	3.66 (3.38, 3.96)	***	3.29 (3.02, 3.58)	***	5.44 (4.74, 6.25)	***	4.86 (4.18, 5.64)	***
Poor	5.24 (4.71, 5.82)	***	4.52 (4.03, 5.06)	***	10.42 (8.93, 12.17)	***	8.88 (7.52, 10.49)	***
Occupational noise exposure duration^g^								
0 year	Ref		Ref		Ref		Ref	
> 0 to 4 years	1.36 (1.29, 1.44)	***	1.23 (1.16, 1.3)	***	1.61 (1.47, 1.75)	***	1.36 (1.24, 1.49)	***
5 to 10 years	1.57 (1.46, 1.70)	***	1.36 (1.26, 1.47)	***	2.19 (1.97, 2.44)	***	1.76 (1.57, 1.97)	***
11 to 15 years	1.71 (1.52, 1.92)	***	1.43 (1.27, 1.61)	***	2.27 (1.95, 2.64)	***	1.76 (1.50, 2.07)	***
> 15 years	1.83 (1.67, 1.99)	***	1.5 (1.37, 1.64)	***	2.50 (2.24, 2.78)	***	1.88 (1.68, 2.11)	***

^a^Odds ratios (ORs) and corresponding 95% confidence intervals (CIs) were estimated using weighted logistic regression for each listed variable, with each model adjusted for age. Participants who selected ‘I prefer not to answer’ were included in the models but not shown in the table

^b^ORs and corresponding 95% CIs were estimated using weighted logistic regression across all listed variables. Participants who selected ‘I prefer not to answer’ were included in the models but not shown in the table

Significant levels: •*p* < 0.1, *p < 0.05, **p < 0.01, ***p < *0.001*

^c^"Other" includes participants who identify as a different race/ethnicity or as multiracial

^d^In the AHS demographic survey, participants rated their socioeconomic status using the MacArthur Scale of Subjective Social Status, a ladder scale from 0 to 9 in the U.S., where 0 represents the "best off"—those with the most money, most education, and most respected jobs—and 9 represents the "worst off”

^e^ “other” includes all the other U.S. territories

^f^Participants were asked, “How would you rate your hearing ability?” with response options: “Excellent”, “Very good”, “Good”, “Fair”, “Poor”, and “I prefer not to answer”

^g^Participants who answered “Yes” to working in a loud workplace were then asked, “How many years have you worked in a loud workplace?” with response options: Less than 5 years, 5–10 years, 11–15 years, More than 15 years, and I prefer not to answer

Compared to non-Hispanic Whites, non-Hispanic Blacks, Hispanics and non-Hispanic Asians had much lower AORs for any and bothersome tinnitus. In both age-adjusted and multivariable models, males had higher odds of any tinnitus (ORs: 1.11 and 1.15) than females, while odds for bothersome tinnitus were similar or slightly lower (ORs: 1.02 and 0.98).

Self-rated hearing ability was the largest independent risk factor for both types of tinnitus. Specifically, participants who rated their hearing as poor had an AOR of 5.24 (95% CI: [4.71–5.82]) for any tinnitus and 10.42 (95% CI: [8.93–12.17]) for bothersome tinnitus, compared to those who rated their hearing as excellent. These differences persisted when controlling for all sociodemographic characteristics and risk factors (MORs of 4.52 and 8.88, respectively). Individuals with prior occupational noise exposure had higher odds of either type of tinnitus than those without exposure (MORs ranging from 1.23 to 1.88). Individuals exposed more than 15 years had the highest odds (any tinnitus MOR: 1.5, 95% CI: 1.37–1.65; bothersome tinnitus MOR: 1.88, 95% CI: 1.68–2.11).

### Weighted decision tree

**Fig. 2:**
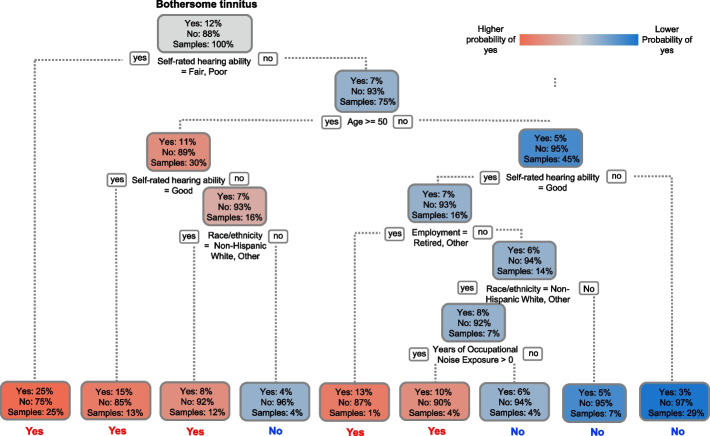
Weighted decision tree identifying predictors of bothersome tinnitus. The weighted decision tree illustrates the strongest predictors of bothersome tinnitus among U.S. adults in the Apple Hearing Study. Age, self-rated hearing ability, employment status, race/ethnicity, and years of occupational noise exposure emerged as key determinants following tree pruning. Each node displays the predicted probability of bothersome tinnitus (“Yes”), the probability of non-bothersome tinnitus or no tinnitus (“No”), and the proportion of participants in that node (“Samples”). Node colors indicate the predicted probability of bothersome tinnitus, with darker red representing higher probabilities and darker blue representing lower probabilities. All 125,252 participants and all listed sociodemographic and risk factors were included in the model. ‘I prefer not to answer’ responses were incorporated in the modeling but are not shown in the figure due to limited interpretability and space constraints

The weighted decision tree model used bothersome tinnitus (yes/no) as the dependent variable and included all sociodemographic and risk factors. The final pruned tree (Fig. 2) identified self-rated hearing ability, age, employment status, race/ethnicity, and years of occupational noise exposure as the strongest risk factors for bothersome tinnitus. The overall likelihood of bothersome tinnitus was 12%. Self-rated hearing ability was the most influential factor: participants with fair or poor hearing had a higher likelihood (25%) of reporting bothersome tinnitus than other hearing ability levels. Among those with better hearing, age further differentiated risk: older adults (≥ 50 years) had a 11% likelihood compared to 5% among younger participants. For individuals with very good or excellent hearing, race/ethnicity, employment status, and occupational noise exposure further stratified risk, with the highest likelihood (13%) observed among retired younger participants (< 50 years) and the second highest (10%) among non-retired Non-Hispanic Whites with ≥ 1 year of occupational noise exposure. The model achieved an AUC of 0.735 (95% CI: 0.727–0.744), an accuracy of 0.632 (95% CI: 0.627–0.637), a sensitivity of 0.76, and a specificity of 0.62. These results were comparable to the performance of the multivariable logistic regression model incorporating all sociodemographic and risk factors (see Additional file 3). The weighted decision tree identified key predictors of bothersome tinnitus with an AUC of 0.735, indicating moderate discrimination. This model is intended as an exploratory tool rather than a clinical decision-making instrument.

## Discussion

Our nationwide cross-sectional analysis of 125,252 volunteer U.S. adults estimated that 3 in 10 adults experience “any tinnitus”, i.e. tinnitus lasting longer than a few minutes on a regular basis, and that 1 in 10 experience “bothersome tinnitus”, i.e. characterized by tinnitus that was frequent, loud, and/or interfered with hearing in the past two weeks. The strongest independent risk factor for tinnitus was self-rated hearing ability. Age-adjustment did not significantly alter this finding. Age was the second most influential independent risk factor, with the MORs of both types of tinnitus increasing with age and peaking between 60–64 years. Race/ethnicity was an important sociodemographic factor, with non-Hispanic Whites having the highest odds of both types of tinnitus. Additionally, years of occupational noise exposure was a strong risk factor. However, neither traditional regression models nor decision tree analyses provided strong predictive power, suggesting that other factors linked to tinnitus were not captured in our models.

Estimated prevalences of any tinnitus (30.8%) and bothersome tinnitus (11.6%) are within the global range of 4.1% to 37.2% reported between 1972–2021 [15]. The prevalence of any tinnitus was closer to the higher end, likely due to our inclusive definition of a tinnitus episode as “a few minutes to an hour” or more (Table 2) as compared to the more restrictive definition of "last more than 5 min" [13, 15]. Prevalence estimates for bothersome tinnitus in this study are slightly higher than the national prevalence rates of 9.6% and 11.2% estimated using data from the U.S. National Health Interview Survey (NHIS) in 2007 and 2014, respectively [18, 28], where tinnitus was defined as lasting > 5 min in the last 12 months and being 'bothersome'. However, the higher prevalence in our study may just reflect an increase in tinnitus due to population aging [29]*.*

Fair or poor self-rated hearing ability was the strongest independent risk factor for both any and bothersome tinnitus. The independence of self-rated hearing ability was further confirmed in the decision tree, as subpopulations with additional risk factors (i.e., older age) did not have a substantially higher likelihood of bothersome tinnitus. This agrees with a previous study that showed tinnitus prevalence was highest (78.6%) among those with severe impairment (65–79.9 dB HL) and lower (42.9%) at complete impairment (≥ 95 dB HL) [12], and that age did not significantly affect tinnitus burden when accounting for hearing impairment. These results highlight the importance of hearing impairment as a predictor of tinnitus. Some tinnitus patients with normal audiograms may nevertheless exhibit notched hearing thresholds [30], which can lower their self-rated hearing ability [31]. When direct measures of hearing impairment are unavailable, combining age with self-rated hearing ability may provide an effective tool for predicting bothersome tinnitus.

Tinnitus odds were higher among older adults after controlling for self-reported hearing ability, occupational noise exposure, sex, and other sociodemographic characteristics. To completely explain away our observed association between age and tinnitus, an unmeasured confounder with approximate odds ratio of 2.01 for any tinnitus and 2.72 for bothersome tinnitus would suffice [32]. Given that participants with more than 15 years of occupational noise exposure had an odds ratio of 1.50 and 1.88 for any and bothersome tinnitus, respectively, and that more than 100 million Americans are exposed to environmental noise levels capable of causing hearing loss [33], noise exposure is a potentially important confounder that could potentially explain the observed age-association [7, 12]. Regardless, our analysis revealed that older adults have much higher odds of bothersome tinnitus (2.72) and any tinnitus (2.01) when compared to the lowest age group (aged 18–24). This increase in burden of tinnitus with age has also been shown in other studies [15].

Tinnitus risk varied among racial/ethnic groups, independent of age and self-rated hearing ability. Non-Hispanic Whites had a higher prevalence of both any and bothersome tinnitus compared to non-Hispanic Blacks, Hispanics, and non-Hispanic Asians, and individuals of other ethnicities (including multiracial). These findings align with previous research on U.S. adults [7, 18, 28]. It is not clear why racial/ethnic minorities had lower tinnitus prevalence. One possible explanation is the often-reported observation that racial/ethnic minorities also have a lower prevalence of hearing loss [34, 35]. This may be partially attributed to differences in skin pigmentation, as more melanocytes may be protective against hearing loss [30, 35].

The association between occupational noise exposure and tinnitus is well-documented [7, 36, 37], but studies including duration of exposure are limited. The multivariable logistic regression model showed that years of occupational noise exposure was associated with tinnitus even after accounting for age and self-rated hearing ability. The odds of bothersome tinnitus were similar among those with 5–10 years (MOR: 1.76) and more than 15 years (MOR: 1.88) of occupational noise exposure, using individuals with no exposure as the reference. This is consistent with prior research indicating that most hearing damage occurs within the first 10 years of occupational noise exposure [38, 39], and highlights the importance of controlling occupational noise exposure for hearing loss and tinnitus prevention.

Although males demonstrated a higher prevalence of tinnitus compared with females, the observed odds ratios indicated a relatively weak association. Given the mixed findings reported in the existing literature [17], the relationship between sex and tinnitus remains inconclusive.

The consistency of our findings with prior population-based studies supports the feasibility of using app-based digital platforms for large-scale epidemiological research. The substantial increase in sample size — enabled by this novel recruitment approach — also enhances statistical power and precision in subgroup analyses.

### Study limitations and future directions

The Apple Hearing Study was primarily designed to examine hearing-related behaviors, environmental sound exposure, and headphone use. Tinnitus was a secondary component, limiting the measures available to define “any” and “bothersome” tinnitus and to evaluate associated risk factors. To address sociodemographic biases, we applied population weighting to improve sample representativeness, although this cannot fully eliminate study limitations. Our study has the typical limitations of self-reported data, including recall and reporting biases, as well as potential self-selection bias, since participants were iPhone users and likely already interested in hearing health, which may lead to under- or overestimation of tinnitus prevalence compared to the general U.S. adult population. This may limit the generalizability of our findings.

Additional limitations include the cross-sectional nature of our analysis, which precludes causal inference. The measured non-response to the five tinnitus questions was very low (< 1%), suggesting minimal bias from respondents; however, potential bias from unmeasured non-respondents remains a limitation. In this analysis, hearing impairment was assessed via a single self-rated question rather than objective audiometry. While this may not fully capture the true variability in hearing loss, self-rated hearing can detect subtle or hidden deficits relevant to tinnitus and aligns with trends observed in studies using objective measures [31]. Finally, neither our logistic regression models nor decision trees indicated strong predictive capabilities, suggesting that there exist additional influential risk factors, such as stress and leisure-time noise exposure, for tinnitus that should be considered in future studies.

A related challenge lies in the variability of tinnitus prevalence across studies, which is influenced not only by differences in study populations but also the lack of standardized definitions for tinnitus [13]. In our study, we defined tinnitus as “at least a few times a year and it lasts a few minutes to an hour” or more for a typical tinnitus episode, attempting to align with the commonly used definition of “last more than 5 min in the last 12 months” [18, 40]. Despite that, our study resulted in higher estimates of bothersome tinnitus (11.6%) prevalence compared to estimated U.S. prevalence levels based on NHIS data in 2007 (9.6%) and 2014 (11.2%) [18, 28].

## Conclusion

This analysis of a large-scale nationwide cohort confirms that tinnitus is prevalent in the U.S., with about one in three adults experiencing any tinnitus and one in ten experiencing bothersome tinnitus. Self-rated poor or fair hearing ability was the strongest independent risk factor for tinnitus, while additional differences were observed based on age, race/ethnicity and occupational noise exposure. However, these factors alone were insufficient to provide strong predictive power for tinnitus, highlighting the importance of consideration of additional potential risk factors in future studies of tinnitus characteristics, including mental health (e.g., anxiety, depression), smoking, sleep disturbances, environmental and recreational noise and migraines.

## Supplementary Information

Supplementary Material 1.

Supplementary Material 2.

Supplementary Material 3.

## Data Availability

Aggregated, de-identified data that support the finding of this study may be available upon request from the corresponding author (RLN). All requests will be reviewed and considered in accordance with policies designed to protect participant confidentiality and to remain consistent with the study protocol and the consent provided by participants.

## Abbreviations

AHS: Apple Hearing Study
CI: Confidence intervals
AOR: Age-adjusted odds ratio
MOR: Multivariable-adjusted odds ratio
AUC: Operating characteristic curve
dB: Decibel
HL: Hearing level
NHIS: National Health Interview Survey
NHANES: National Health and Nutrition Examination Surveys
