# Supplementary material for: Tinnitus prevalence and characteristics in the United States: insights from a cross-sectional analysis of the 2019–2022 Apple Hearing Study cohort
Source: BMC Public Health. 2026 Mar 19;26:1385. doi: 10.1186/s12889-026-27048-2 (PMC13122993; doi:10.1186/s12889-026-27048-2)
Supplement: Supplementary file 1 — Supplementary Material 1. [file 12889_2026_27048_MOESM1_ESM.docx]

# Survey Questions

## Tinnitus Questions

**How often do you hear tinnitus?**

Tinnitus can sound like ringing, whistling, buzzing, or roaring in your ears. (choose one only)

- Never
- Up to a few times a year
- Up to a few times a month
- Up to a few times a week
- Up to a few times a day
- I prefer not to answer

If the answer is not never, proceed to next question.

**How long does your typical episode of tinnitus last?**

(choose one only)

- Less than a few seconds
- A few seconds to a minute
- A few minutes to an hour
- Hours to days
- It is constant
- I prefer not to answer

If the answer is not never, proceed to next question.

**Over the past two weeks, how often were you aware of tinnitus?**

(choose one only)

- Always
- Very frequently
- Occasionally
- Rarely
- Never
- I prefer not to answer

If the answer is not never, proceed to next question.

**How loud was the tinnitus?**

(choose one only)

- Extremely loud
- Very loud
- Moderately loud
- Not very loud
- Barely noticed
- I prefer not to answer

If the answer is not never, proceed to next question.

**Over the past two weeks, how much has tinnitus interfered with your ability to hear clearly?**

(choose one only)

- Completely
- Moderately
- Slightly
- Not at all
- I prefer not to answer

** The questions in the tinnitus section were adapted from the Tinnitus Functional Index (TFI), a visual analog scale (VAS) for tinnitus loudness.*

## Sociodemographic Questions

**What was the sex assigned on your original birth certificate?**

(choose one only)

- Female
- Male
- Intersex
- I prefer not to answer

**Which categories describe you?**

This question is from the All of Us study, a large research program sponsored by the National Institutes of Health (NIH).

(Select all that apply)

- American Indian or Alaska Native
- Asian
- Black, African American, or African
- Hispanic, Latino, or Spanish
- Middle Eastern or North African
- Native Hawaiian or other Pacific Islander
- White
- None of these fully describe me
- I prefer not to answer

**What is the highest grade or year of school you completed?**

This question was adapted from the Behavioral Risk Factor Surveillance System, an annual survey conducted by the US Centers of Disease Control (CDC).

(choose one only)

- Never attended school or only attended kindergarten
- Grades 1 through 4 (Primary)
- Grades 5 through 9 (Middle school)
- Grades 9 through 11 (Some high school)
- Grades 12 or GED (High school graduate)
- 1 to 3 years after high school (Technical school)
- 1 to 3 years after high school (Some college, Associate’s Degree)
- College 4 years or more (College graduate)
- Advanced degree (Master’s Degree)
- Advanced degree (Doctorate Degree)
- I prefer not to answer

**Which of the following best describes your current employment situation?**

This question was adapted from the U.S. Census Current Population Survey.

(choose one only)

- Employed for pay (part-time, full-time, self-employed)
- Unemployed
- Unable to work (i.e., disability, illness, other circumstances)
- In school
- Taking care of house or family
- In retirement
- I prefer not to answer

**Are you…**

This question was adapted from the Behavioral Risk Factor Surveillance System, an annual survey conducted by the US Centers of Disease Control (CDC).

(choose one only)

- Married
- Divorced
- Widowed
- Separated
- Never married
- A member of an unmarried couple
- I prefer not to answer

**Where would you place yourself on this ladder?**

This question is the MacArther Scale of Subjective Social Status, which is a standardized questions that research has shown is related to overall health.

Think of this ladder as representing where people stand in the country you live in.

At the top of the ladder are the people who are the best off – those who have the most money, the most education, and the most respected jobs. At the bottom are the people who are the worst off – those who have the least money, least education, the least respected jobs, or no job. The higher you are up on this ladder, the closer you are to the people at the very top; the lower you are, the closer you are to the people at the very bottom.

Please select where you think you stand at this time in your life relative to other people around you.

(choose one only)

- Worst off, 9
- Prong , 8
- Prong, 7
- Prong , 6
- Prong , 5
- Prong , 4
- Prong , 3
- Prong , 2
- Prong, 1
- Best off , 0

**What is the population of the city or town you live in?**

(choose one only)

- More than 1 million people
- 300,001 – 1 million
- 100,001 - 300,000
- 10,001 – 100,000
- 1,000 – 10,000
- less than 1,000
- I prefer not to answer

## Hearing and Environmental Exposure Questions

**Including you, how many people live in your household?**

(choose one only)

- One
- Two
- Three
- Four
- Five or more
- I prefer not to answer

**How would you rate your hearing ability?**

(choose one only)

- Excellent
- Very good
- Good
- Fair
- Poor
- I prefer not to answer

**Do you now or have you ever worked in a loud workplace?**

If you had or have to raise your voice to be heard by someone standing an arm's length away, the answer is yes.

(choose one only)

- Yes
- No
- I don’t know
- I prefer not to answer

If the answer is not never, proceed to next question.

**How many years have you worked in a loud workplace?**

(choose one only)

- Less than 1 year
- 1-2 years
- 3-4 years
- 5-10 years
- 11-15 years
- 16-20 years
- More than 20 years
- I prefer not to answer

* Age was calculated based on the birth year provided by participants. Regions were defined according to the U.S. Census Bureau’s “Census Regions and Divisions of the United States” (<https://www2.census.gov/geo/pdfs/mapsdata/maps/reference/us_regdiv.pdf>), using participants’ state information. Both age and state were collected at the time of consent.
