# Supplementary material for: Tinnitus prevalence and characteristics in the United States: insights from a cross-sectional analysis of the 2019–2022 Apple Hearing Study cohort
Source: BMC Public Health. 2026 Mar 19;26:1385. doi: 10.1186/s12889-026-27048-2 (PMC13122993; doi:10.1186/s12889-026-27048-2)
Supplement: Supplementary file 2 — Supplementary Material 2. [file 12889_2026_27048_MOESM2_ESM.docx]

**Flowchart for participants selection.**


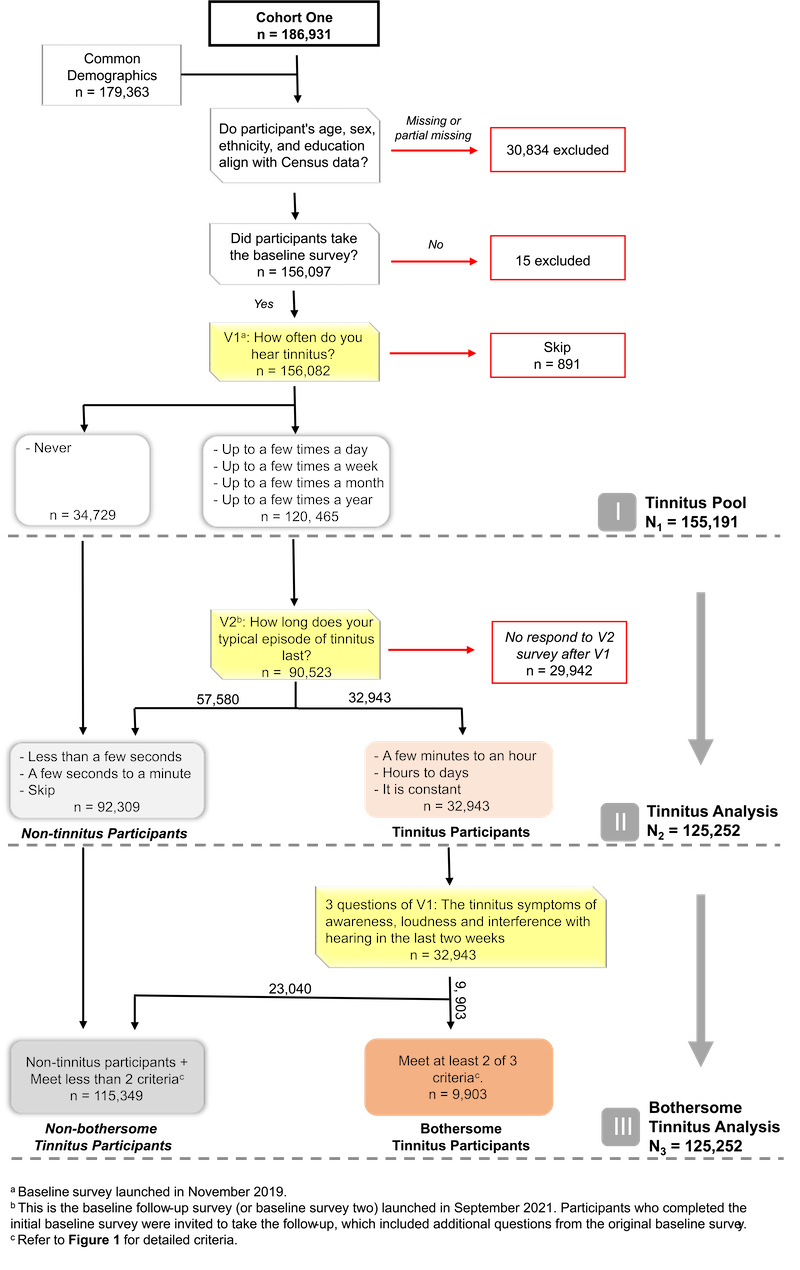


^a^ Baseline survey launched in November 2019.

^b^ This is the baseline follow-up survey (or baseline survey two) launched in September 2021. Participants who completed the initial baseline survey were invited to take the follow-up, which included additional questions from the original baseline survey.

^c^ Refer to **Figure 1** for detailed criteria.
