# Supplementary material for: Tinnitus prevalence and characteristics in the United States: insights from a cross-sectional analysis of the 2019–2022 Apple Hearing Study cohort
Source: BMC Public Health. 2026 Mar 19;26:1385. doi: 10.1186/s12889-026-27048-2 (PMC13122993; doi:10.1186/s12889-026-27048-2)
Supplement: Supplementary file 3 — Supplementary Material 3. [file 12889_2026_27048_MOESM3_ESM.docx]

| **Model performance of the weighted multivariable logistic regression of bothersome tinnitus** | |
| --- | --- |
| **Metrics** | **Values** |
| Area under the curve (AUC), CI 95% | 0.771 (0.755, 0.787) |
| Accuracy, CI 95% | 0.711 (0.703, 0.719) |
| Sensitivity | 0.706 |
| Specificity | 0.712 |
| Model performance was evaluated using weighted data from 125252 participants in the Apple Hearing Study. Metrics include the area under the receiver operating characteristic curve (AUC), sensitivity, specificity, and overall accuracy. The model incorporated all assessed sociodemographic and risk factors. | |
